# Management of Cardiovascular Disease in Patients With COVID-19 and Chronic Chagas Disease: Implications to Prevent a Scourge Still Larger

**DOI:** 10.3389/fmed.2022.910388

**Published:** 2022-06-29

**Authors:** Reinaldo Bulgarelli Bestetti, Edimar Alcides Bocchi, Renato Bestetti, Victor Sarli Issa, Rosemary Aparecida Furlan-Daniel, Marcelo Arruda Nakazone

**Affiliations:** ^1^Department of Medicine, Medical School, University of Ribeirão Preto, Ribeirao Preto, Brazil; ^2^Heart Institute, University of São Paulo, Saõ Paulo, Brazil; ^3^Department of Cardiology, Antwerp University Hospital, Antwerp, Belgium; ^4^Department of Cardiology and Cardiovascular Surgery, Faculdade de Medicina de São José do Rio Preto, São José do Rio Preto, Brazil

**Keywords:** COVID-19, Chagas disease, cardiovascular disease, SARS-CoV-2, *Trypanosoma cruzi*

## Abstract

Cardiovascular diseases (CVD) are the most important cause of morbidity and mortality in the general population. Because the high prevalence of COVID-19 and chronic Chagas disease (CCD) where the latter is endemic, all such diseases will likely be observed in the same patient. While COVID-19 can provoke generalized endotheliitis, which can lead to a cytokine storm and a hyper-coagulable state culminating into in-site and at a distance thrombosis. Therefore, small-vessel coronary artery disease (CAD), cerebrovascular disease, thromboembolism, and arrhythmias are prominent findings in COVID-19. In CCD, small-vessel CAD, cardioembolic stroke, pulmonary embolism, heart failure and arrhythmias are frequently observed as a result of a similar but less intense mechanism. Consequently, the association of CCD and COVID-19 will likely increase the incidence of CVD. Thus, doctors on the frontline should be on the alert for this diagnostic possibility so that the proper treatment can be given without any delay.

## Introduction

At the time of this writing, the COVID-19 pandemic has affected more than 312 million people, and ceased life of 5.5 million people worldwide, with a mortality rate approaching 1.76% (1). COVID-19 is caused by SARS-CoV-2, and can unmask, aggravate, or provoke cardiovascular disease (CVD), including acute coronary syndrome, cerebrovascular disease (CEVD), acute heart failure (AHF) [acute decompensating of chronic heart failure (CHF) or *de novo* heart failure], thromboembolism, arrhythmias, and sudden cardiac death (SCD).

CVD are the main cause of morbidity, mortality, and disability-adjusted life years (DALY) globally. The economic burden of CVD will be USD 1,044 billion by 2030. Furthermore, loss of productivity due to morbidity and mortality accounts for 11 and 15%, respectively, of total healthcare costs. Finally, the prevalence of DALY associated with CVD is 4,530/100,000 inhabitants (2).

Chagas disease is caused by the protozoan *Trypanosoma cruzi*, which is transmitted in the majority of cases by a kissing bug. After the initial infection, which terminates spontaneously in the overwhelming majority of cases, approximately 60% of patients are asymptomatic, showing only a positive serology. They are said to be in the indeterminate stage of the disease. However, about 20–30% of patients can develop chronic Chagas heart disease (CCHD) up to 20 years after initial infection (3).

**Figure 1:**
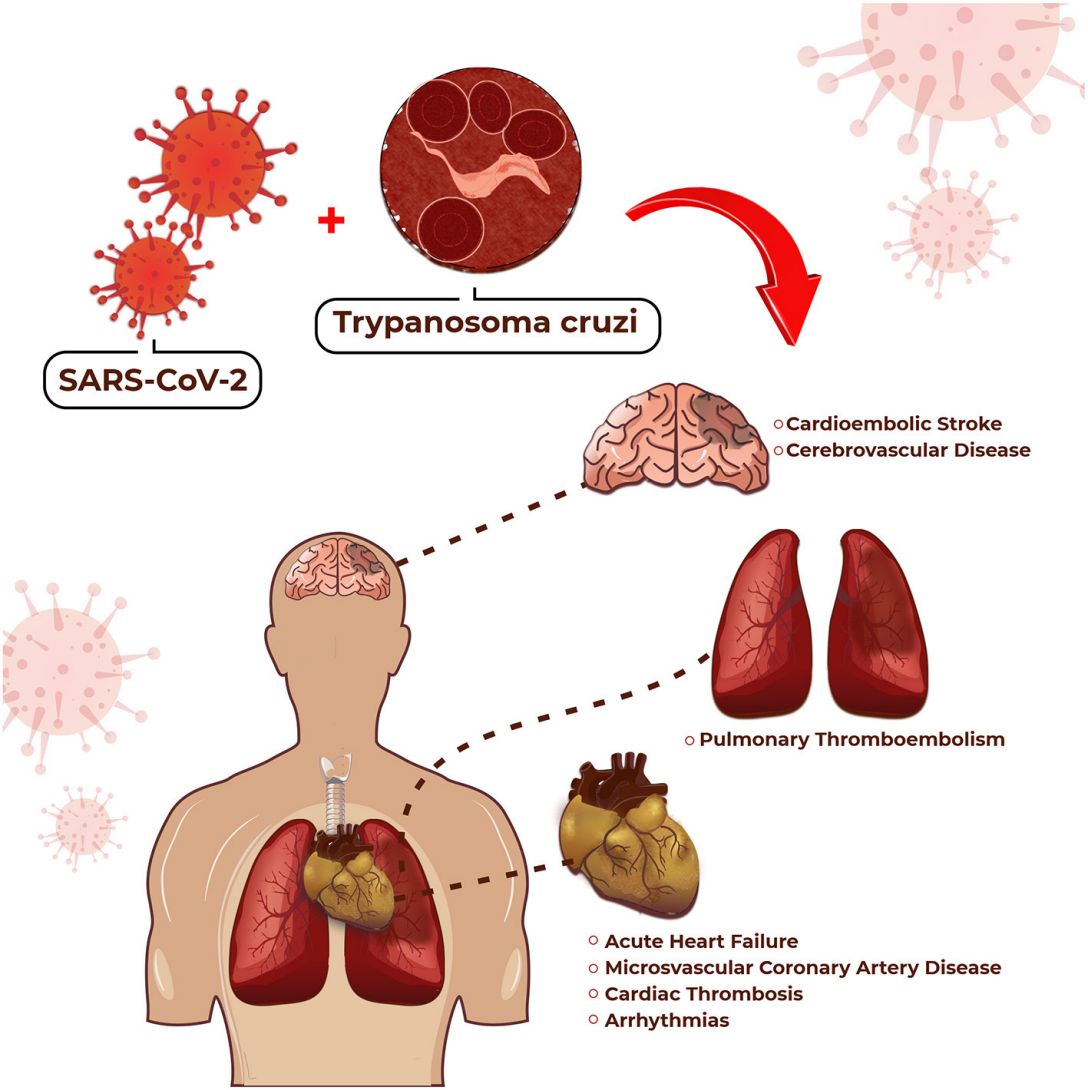
Potential cardiovascular complications in patients with CCHD and COVID-19.

CCHD manifests as AHF or CHF, thromboembolic phenomena, cardiac arrhythmias (3), and obstructive or non-obstructive coronary artery disease (CAD) (4). Therefore, the management of patients with CVD with superimposed COVID-19 in an area where Chagas disease is endemic is a colossal task because all diseases may manifest at the same time in the same patient. The potential cardiovascular complications for which we should be on the alert in the association of CCHD and COVID-19 are highlighted in Figure 1. On the other hand, Table 1 compares some particularities between these manifestations.

**Table 1:** Cardiovascular implications in patients with Chagas disease and COVID-19.

**Chronic Chagas disease**	**COVID-19**
**Coronary artery disease**	
• Caused by endothelial dysfunction secondary to traditional cardiovascular risk factors and several comorbidities, including dyslipidemia, diabetes mellitus, systemic arterial hypertension, and smoking. • Thrombus and coronary spasm even in the angiographically proven normal epicardial coronary arteries are well other recognized causes of CAD.	• In patients with COVID-19 the mechanism behind CAD clinical manifestations may be different of that seen in non-COVID-19 patients, suggesting a major hypercoagulation state. • SARS-CoV-2 can recruit macrophages, which secrete collagenases in the endothelium, thus destroying the fibrous cap and leading to plaque rupture and intraluminal thrombosis.
**Microvascular disease**	
• Abnormalities in the microcirculation caused by vasospasm and platelet aggregation associated with inflammation and autonomic dysfunction have been postulated. • Acute coronary syndrome with non-obstructive coronary arteries can be associated with this condition. Moreover, microvascular disease-induced ischemia can progress to myocardial fibrosis and ventricular remodeling in patients with CCHD.	• In patients with COVID-19, the potential for the occurrence of abnormalities in the epicardial and small coronary arteries does exist. • Usually, acute coronary syndromes occur in patients with previously known/unknown CAD. Patients should undergo coronary angiography without any delay; the absence of CAD prompts the diagnosis of microvascular angina.
**Heart failure**	
• The reason of the dismal outcome in patients with CHF due to CCHD may be ascribed to the aggressive ventricular remodeling, secondary to association of mononuclear cell infiltrate and reparative fibrosis throughout the myocardium. • AHF in patients with Chagas disease should promptly be diagnosed because the very poor prognosis associated with this condition.	• Individuals with advanced CHF do not necessarily develop fever or other signs consistent with acute infection syndrome, thus suggesting that acute decompensation of CHF can be caused by COVID-19. • Patients with previous compensated CHF coinfected with SARS-CoV-2 are at higher risk of developing AHF than patients without.
**Cerebrovascular disease**	
• CHF, mural thrombus, left ventricular apical aneurysm, and cardiac arrhythmias (particularly atrial fibrillation) are independent predictors of cardioembolic stroke and CEVD in patients with CCD.	• The risk of both ischemic stroke and cryptogenic stroke in COVID-19 patients is four times higher than that observed in non-COVID-19 patients, despite comorbidities.
**Cardiac thrombosis and pulmonary embolism**	
• Cardiac thrombosis might be the consequence of generalized vasculitis, as part of a chronic inflammation, culminating in platelet hyper aggregation, which has been detected even in patients in the indeterminate stage of CCD. • The high frequency of PE in patients with CCHD appears to be the consequence of a high prevalence of right-sided cardiac thrombosis-induced PE rather than *in situ* thrombosis of the affected areas.	• The SARS-CoV-2 binds to the angiotensin converting enzyme 2 receptor of vascular endothelial cells in the heart microvasculature, thus provoking endothelial activation; which releases several proinflammatory factors and lead to platelet aggregation and superimposed thrombosis. • PE in patients with COVID-19 has been associated with coagulopathy and inflammation leading to intrapulmonary thrombosis, rather than with traditional deep-vein thromboembolism.
**Cardiac arrhythmias and sudden cardiac death**	
• Non-sustained ventricular tachycardia appears to be 30 times more frequent than in those with non-Chagas CHF. Approximately 10% of patients with CCHD with this arrythmia subsequently present sustained ventricular tachycardia and may degenerate into ventricular fibrillation in 24% of cases. • Macro re-entry at the His-Purkinje system can be the cause of SCD in patients with CCHD. However, myocardial fibrosis appears to play a central role in the pathogenesis of SCD in patients with CCHD.	• Several abnormalities underlie the appearance of arrhythmias in hospitalized patients with COVID-19, such are hypoxia, acid-base imbalance, electrolyte disturbance, and myocardial ischemia. • Therefore, malignant arrhythmia *per se* does not seem to be the etiology of death in patients with critical or severe COVID-19.

*AHF, acute heart failure; CAD, coronary artery disease; CCD, chronic Chagas disease; CCHD, chronic Chagas heart disease; CEVD, cerebrovascular disease; CHF, chronic heart failure; COVID-19, coronavirus disease 2019; PE, pulmonary embolism; SARS-CoV-2, severe acute respiratory syndrome coronavirus 2; SCD, sudden cardiac death*.

The aim of this paper is to provide suggestions for the management of CVD in patients with COVID-19 in areas where Chagas disease is endemic because coinfection—Chagas disease and SARS-CoV-2- may further aggravate morbidity, mortality, and DALY of patients with CVD.

## Cardiovascular Diseases Management

### Coronary Artery Disease

The incidence of CAD is 176.3/100,000 inhabitants, whereas the prevalence of this disease is 2,270/100,000 inhabitants (2). CAD is the most important cause of death in patients with CVD (5), accounting for 38% of overall deaths (2). In addition, the DALY associated with CAD approaches 54% of the total DALYS caused by CVD (2). The economic burden of CAD is substantial, as it is associated with 27% of the total costs of CVD (2).

CAD is initially caused by endothelial dysfunction secondary to several comorbidities, including dyslipidemia, diabetes mellitus, systemic arterial hypertension, and smoking. At first, the low-density lipoprotein cholesterol fraction penetrates the intima of the dysfunctional endothelium; inflammatory cells are recruited to the vascular intima, and ingest low-density lipoprotein cholesterol fraction, thus forming the foam cells and inducing the proliferation of vascular smooth cells. The latter forms a fibrous cap and, ultimately, an established atherosclerotic plaque ensues. The narrowing luminal provoked by plaque or plaque rupture-associated thrombus superposition are the main mechanisms behind the clinical manifestations of CAD (5). However, thrombus and coronary spasm even in the angiographically proven normal epicardial coronary arteries are well other recognized causes of CAD.

Clinically, CAD manifests by stable angina pectoris or acute coronary syndrome—unstable angina pectoris, and acute myocardial infarction with elevated ST-segment (STEMI) or non-elevated ST-segment (non-STEMI). Nonetheless, many patients are found to have silent CAD, and they are at risk of death as well.

In patients with COVID-19, STEMI incidence may have increased by 35%, suggesting that SARS-CoV-2 infection itself might have caused such abnormality (6). In fact, SARS-CoV-2 recruits macrophages, which secrete collagenases in the endothelium, thus destroying the fibrous cap and leading to plaque rupture and intraluminal thrombosis (7). Therefore, SARS-CoV-2 infection may induce episodes of acute coronary syndrome in patients with CAD. In addition, a case of severe vasospasm has also been detected in a patient with COVID-19 (8). Furthermore, in patients with COVID-19 and STEMI, as compared with patients with STEMI without COVID-19, a higher proportion of multiple culprit thrombotic lesions, stent thrombosis, higher thrombus grade, and more importantly, lower myocardial blush grade following procedural intervention compatible with microthrombi have been observed (9).

Overall, angiographic studies show that about one-third of patients with STEMI and COVID-19 do not have underlying obstructive CAD. There are parallel autopsy studies in which cardiac thrombosis is found in 79% of patients with myocardial necrosis. However, underlying epicardial coronary thrombosis is detected in only 14% of patients; the remaining individuals are found to have microthrombi in myocardial capillaries, arterioles, and small muscular arteries (6). Collectively, such findings suggest that in patients with COVID-19 the mechanism behind CAD clinical manifestations may be different from that seen in non-COVID-19 patients, suggesting a major hypercoagulation state. This can be found in patients with chronic Chagas disease (CCD) as well.

Chest pain—atypical or typical—can be found in up to 15% of patients with CCD (10). Megaoesophagus has been associated with chest pain in patients with this condition (11). Nonetheless, in the absence of concomitant megaoesophagus, microvascular CAD seems to underlie chest pain in patients with CCHD (12), as obstructive epicardial CAD is rarely seen in patients with this condition (13, 14). Moreover, abnormalities in the tonus of epicardial coronary arteries have also been observed in patients with CCHD (15).

The presence of microvascular CAD with or without precordial chest pain can impact on the clinical course of patients with CCHD. In fact, acute coronary syndrome can be associated with this abnormality (10), and atypical chest pain can herald the appearance of acute myocardial infarction with non-obstructive coronary arteries in patients with such a condition (4). Moreover, microvascular disease-induced ischemia can progress to myocardial fibrosis and ventricular remodeling in patients with CCHD (16).

The pathophysiology of microvascular CAD in patients with CCD is not fully understood at this time; however, abnormalities in the microcirculation caused by vasospasm and platelet aggregation associated with inflammation and autonomic dysfunction have been postulated (17, 18). In this regard, a 3-month treatment with verapamil (80 mg/bid) and acetylsalicylic acid (100 mg/once a day) reduced the burden of ischemic episodes in patients with CCHD with microvascular CAD (19).

In patients with COVID-19 and CCHD, the potential for the occurrence of abnormalities in the epicardial and small coronary arteries does exist. Such abnormalities, according to all that have previously been mentioned, will more likely be manifested by acute coronary syndromes, either in patients with previously known CAD or in patients with unknown CAD. Therefore, such patients should undergo coronary angiography without any delay; if obstructive CAD is found, patients should be treated according to guidelines. However, if non-obstructive CAD is the diagnosis, calcium inhibitors and acetylsalicylic acid should be considered. In the case of chronic chest pain, myocardium scintigraphy or stress echocardiography should be performed. If ischemia is detected, patients should undergo coronary angiography; the absence of CAD prompts the diagnosis of microvascular angina, and calcium inhibitors and acetylsalicylic acid should be considered. If necessary, beta-blockers can be added to the treatment (20).

### Cerebrovascular Disease

The incidence of stroke is about 143/100,000 inhabitants, whereas the prevalence of CEVD is 1,276/100,000 inhabitants. Stroke is the second most important cause of death in patients with CEVD, being responsible for 26% of all CVD deaths. CEVD is the second leading cause of disability, accounting for 27% of all DALYs associated with CVD. Moreover, CEVD impacts 20% on the overall costs on CVD (2).

Overall, about 38% of CEVD is associated with COVID-19 at the present time (6). In patients with COVID-19, the incidence of CEVD can be as high as 4.5% of the affected population (21–23). The prevalence of ischemic stroke (1.1%) is by far more common than haemorrhagic stroke (0.2%) in patients with COVID-19. A neuroimaging study shows that about 60% of cases of haemorrhagic stroke are not associated with systemic arterial hypertension (6). The risk of both ischemic stroke and cryptogenic stroke in COVID-19 patients is four times higher than that observed in non-COVID-19 patients.

The treatment of COVID-19-associated CEVD has become cumbersome during the pandemic. As observed in other diseases, the fear of exposure to COVID-19 in hospital settings probably explains the reduced 31% of reperfusion, and 22% of mechanical thrombectomies in patients with stroke (6). In-hospital mortality is higher (33 vs. 13%) in patients with COVID-19 associated stroke than in patients with stroke not associated with COVID-19 (24). This in-hospital mortality is three times higher than that seen in the pre-pandemic era despite similar door-to-needle time, time to thrombolysis with alteplase, door-to-reperfusion, and time of successful recanalization (25). Collectively, these findings suggest a more aggressive pathophysiological mechanism in patients with CEVD with COVID-19 than in those without. Therefore, the intensive management of patients with CEVD in the setting of COVID-19 is much necessary.

The prevalence of CEVD is 2% in patients with CCD (26). However, CEVD can affect about 11% of patients with CCHD (27), and up to 20% of a referral population of patients with this condition (28). In patients with CCD, CEVD manifests by cardioembolic stroke in up to 76% of cases; nonetheless, 12% of such patients are found to have occlusion of a major cerebral vessel, whereas 6% had occlusion of a small cerebral artery. Haemorrhagic stroke was not found. Risk factors for cardioembolic stroke are diabetes mellitus, young age, female sex, previous stroke, and a Souza score ≥ 3, whereas systemic arterial hypertension and dyslipidaemia are risk factors for non-cardioembolic stroke (29, 30).

CEVD may be the first clinical manifestation of CCHD in 3% to 33% of cases, and stroke recurrence can be found from 3 to 20% of patients (31). CHF, mural thrombus, left ventricular apical aneurysm, and cardiac arrhythmias [particularly atrial fibrillation] are independent predictors of CEVD in patients with CCD (32). In patients with mild to moderate CHF due to CCHD, left ventricular apical aneurysm and left ventricular thrombus are independent predictors of CEVD (28). Stroke is more frequently found in patients with CCHD with CHF than in those without, and the diagnosis of CCD is an independent predictor of stroke in patients with CHF (32).

Such clinical data are supported by a landmark morphological study carried out in 1,345 autopsied patients, which revealed cardiac thrombosis in 370 (28%) and thromboembolism in 638 (57%) patients. In that study, a cardiac thrombus was detected in the left ventricle in 121 (33%) patients; cardioembolic stroke occurred in 21% of patients (33).

Collectively, such data suggest that patients with COVID-19, CCD, and CEVD must be screened for cardioembolic stroke with echocardiography without any delay, and routine anticoagulation should be promptly administered if the diagnosis of cardioembolic stroke is made. In those patients in which cardioembolic stroke has been discarded or is a less likely diagnosis, the protocol for treating non-cardioembolic stroke should be immediately given.

### Heart Failure

The prevalence of CHF is approximately 1–2% of the general population. The incidence of CHF approaches 5/1,000 patients-year in an adult population (34). CHF may affect about 3% of patients with COVID-19 (35). Approximately 23% of hospitalized patients with COVID-19 develop AHF; the prevalence is still higher (49%) in those who died (36). In non-Chagas disease patients with cardiovascular risk factors and no previous history of CHF, a condition similar to that seen in patients in the indeterminate stage of CCD, AHF appears in about 20% of patients with superimposed COVID-19 (37).

Coinfection with SARS-CoV-2 has been associated with death in patients in the indeterminate phase of CCD (38). Therefore, in patients in the indeterminate stage of CCD who develop AHF the diagnosis of COVID-19 should be considered. In this regard, even patients with mild symptoms should be suspected of having COVID-19 coinfection because the SARS-CoV-2 strain that is grassing at the time of this writing (Omicron strain) can manifest a clinical profile different from that seen with the other SARS-CoV-2 strains (39).

AHF in patients with Chagas disease should promptly be diagnosed because the very poor prognosis associated with this condition. A study that included 767 patients, one in five with CCHD, showed that the proportion of cardiogenic shock at hospital admission was higher in Chagas disease than in non-Chagas disease patients. In addition, Chagas disease patients were more frequently in need of inotropic support or intra-aortic balloon pump, and had larger hearts than non-Chagas disease patients. Moreover, prognosis at 6 months were poorer in Chagas disease than in non-Chagas disease patients (40).

Non-Chagas disease patients with previous compensated CHF coinfected with SARS-CoV-2 are at higher risk (3 times more) of developing AHF than patients without; the mortality rate is 48% in patients with CHF and 19% in those without. *De novo* AHF can be detected in about 1.5% of patients with COVID-19. The withdrawal of beta-blockers, angiotensin converting enzyme inhibitors/angiotensin receptors blocks, and mineralocorticoid antagonists are the powerful independent predictors of mortality in such patients (41).

In non-Chagas disease patients with advanced CHF, Bocchi et al. reported that infection with SARS-CoV-2 worsens clinical status, as shown by the increased need for inotropic support or the introduction of a new inotropic drug for cardiac compensation. However, and not unexpectedly, mortality was dramatically increased at the 6-month follow-up. Interestingly, patients with advanced CHF with clinical deterioration following SARS-CoV-2 infection do not necessarily develop fever or other signs consistent with acute infection syndrome, thus suggesting that acute decompensation of CHF can be caused by COVID-19 in a region with high transmissibility of SARS-CoV-2 (42).

CHF can be found in about 14% of patients with CCD in a population-based cohort (43), and in up to 46% of patients in a referral center population (44). CHF is the main clinical manifestation of CCHD and the leading cause of CHF in endemic areas (45). The outcome of CHF secondary to CCHD is relentless, with an annual mortality of up to 20% (46). This high overall mortality may account, at least in part, for the poorer outcome observed in CHF due to CCHD in comparison with other causes of CHF (47–49). The reason for this dismal outcome may be ascribed to the aggressive ventricular remodeling observed in patients with this condition i.e., the association of mononuclear cell infiltrate and reparative fibrosis throughout the myocardium (3).

CHF secondary to CCHD is characterized by reduced left ventricular ejection fraction (LVEF); no cases of CHF due to left ventricular diastolic dysfunction have been observed. Therefore, the treatment of CHF secondary to CCHD is similar to that given to patients with non-Chagas disease heart failure, consisting of beta-blockers, angiotensin converting enzyme inhibitors/angiotensin receptor blocks, and mineralocorticoid antagonists (50). Ivabradine can also be of value to some Chagas disease patients with CHF (51). Reversion of the ventricular remodeling can occur in patients with CHF secondary to CCHD, but its significance on the prognosis of such patients is unknown (52). About 39% of patients with this condition do not tolerate the association of beta-blockers with angiotensin converting enzyme inhibitors/angiotensin receptor blocks at targeted doses because of symptomatic systemic arterial hypotension (53). This fact can also account for the unfavorable outcome of such patients.

Molina et al. screened 7,018 hospitalized patients with COVID-19 from a Brazilian Registry; 31 (0.4%) of them had CCD, and 124 patients with COVID-19 alone (matched by sex, age, systemic arterial hypertension, diabetes mellitus) served as controls. Compared with controls, patients with CCD and COVID-19 had a higher prevalence of AHF and atrial fibrillation, as well as lower levels of C-reactive protein. However, the proportion of patients who progressed to critical COVID-19 and overall mortality were similar in both groups (54). Although the small sample size precludes a firm conclusion regarding the impact of COVID-19 on patients with CCHD, it appears that coinfection—Chagas disease and COVID-19 can precipitate AHF in such patients. Furthermore, coinfection with SARS-CoV-2 can be associated with death in patients with CCHD (38).

This might be the consequence of viral infection itself, which could induce more myocardial inflammation, myocardial necrosis, and myocardial fibrosis. In fact, patients with Chagas cardiomyopathy show an increase in angiotensin converting enzyme 2 serum levels, which are associated with cardiac damage, ventricular remodeling, and unfavorable outcome in patients with CCD (55). Theoretically, therefore, infection with *T. cruzi* might facilitate SARS-CoV-2 binding to angiotensin converting enzyme 2 and start a vicious cycle. Another pathogenetic mechanism could be the overexpression of proinflammatory cytokines, especially interferon-37, which plays a central role in the pathogenesis of CCD (3). Heart endothelial inflammatory dysfunction, a condition observed in both diseases (56, 57), could also play a role in the aggravation of heart disease by provoking microvascular coronary disease, microvascular thrombosis, chronic ischemia, and ventricular remodeling.

In essence, patients in the indeterminate stage of CCD and compensated CHF due to CCHD should follow all restrictive measures in an attempt to avoid the infection of COVID-19 (58). In addition, those with compensated CHF should closely be followed-up to monitor adherence to guideline-recommended treatment because of the negative impact of the non-adherence to treatment on the prognosis of patients with this condition.

### Cardiac Thrombosis and Pulmonary Embolism

The epidemiology of pulmonary embolism (PE) is scanty. The prevalence of PE is estimated to be 60/100,000 inhabitants, while the prevalence of deep vein thrombosis, the major risk factor for PE, is 124/100,000 persons. The incidence of PE is estimated to approach 1/1,000 inhabitants yearly. PE is considered to be the third leading cause of CVD around the western world (59).

The prevalence of PE in patients with COVID-19 can vary from 8 to 16% (60, 61). The prevalence of PE is higher in patients aged < 65 years (20%) than in older patients (14%) (62). C-reactive protein, male gender, and time from symptom onset to hospitalization were positively associated with PE on multivariate analysis (60). Therefore, PE in patients with COVID-19 has been associated with coagulopathy and inflammation rather than with traditional deep-vein thromboembolism; this means that traditional anticoagulant risk factors may not be major determinants of PE in patients with COVID-19 (60).

Clinically, the clinical picture of PE in hospitalized patients with COVID-19 is characterized by the persistence or deterioration of respiratory symptoms, increased oxygen requirement, and a rapid increase in D-dimer levels. Approximately 28% of patients had deep vein thrombosis of the lower limbs, and 43% were on heparin prophylaxis. Chest computed tomography reveals massive pulmonary embolism or thrombosis of pulmonary arteries with segmental of subsegmental pattern; in some patients, subsegmental thrombosis of pulmonary arteries within lung opacities suggested local mechanisms rather than embolism at a distance. Overall mortality is around 1% (63).

Autopsy studies have shown that PE in patients with COVID-19 is associated with pulmonary intravascular coagulopathy leading to intrapulmonary thrombosis (64). In an African autopsy series, PE has been observed in 76% of patients who died suddenly in the community, but only 14% were found to have concomitant venous thrombosis (64). Therefore, a high level of suspicion is necessary to make the diagnosis of PE in patients with COVID-19.

Prophylaxis with anticoagulants in patients with COVID-19 is still evolving. In patients with COVID-19 admitted to the critical care unit, a randomized trial showed that the impact of standard-dose prophylactic anticoagulation with enoxaparin on vascular thrombosis, treatment with extracorporeal membrane oxygenation, or mortality was similar to that observed with intermediate- dose prophylactic anticoagulation. Therefore, intermediate-dose prophylactic anticoagulation appears not to be indicated to treat patients with severe COVID-19 (65). However, in non-critically hospitalized patients with COVID-19, treatment with a therapeutic dose of enoxaparin improved outcome (survival to hospital discharge, reduced need of respiratory or cardiovascular support at discharge) in comparison with usual prophylactic anticoagulation (66). In cases of PE presenting with cardiogenic shock, however, catheter-directed thrombolysis should be performed promptly, as it may be lifesaving (67).

A landmark autopsy study has revealed that right-sided cardiac thrombus can be detected in the right atrium in about 20% of patients with CCHD. PE can be diagnosed in 27% patients; a concomitant right-sided cardiac thrombus was found in 33% of such patients. This suggests that a substantial number of right-sided thrombosis progressed to PE (33).

Clinically, the diagnosis of Chagas disease is an independent predictor of PE (68), and right-sided cardiac thrombosis can be detected in about 58% of patients with Chagas disease heart failure (69). Nevertheless, right-sided cardiac thrombosis can be found in patients with right ventricular dysfunction without overt CHF (70). Therefore, the high frequency of PE in patients with CCHD appears to be the consequence of a high prevalence of right-sided cardiac thrombosis-induced PE rather than *in situ* thrombosis of the affected areas.

The mechanism behind cardiac thrombosis in patients with CCHD is obscure. Cardiac thrombosis might be the consequence of generalized vasculitis as part of a generalized chronic inflammation (71), culminating in platelet hyper aggregation (72), which has been detected even in patients in the indeterminate stage of chronic Chagas disease (73). Some variants of the human platelet polymorphism may be associated with CCHD severity, thus lending credence to the involvement of platelets on the thrombotic process in patients with CCD (74).

The SARS-CoV-2 binds to the angiotensin converting enzyme 2 receptor of vascular endothelial cells, including those placed in the heart microvasculature, thus provoking endotheliitis (36). Endothelial activation releases several proinflammatory cytokines, chemokines, von Willebrand and factor VIII, which lead to platelet aggregation and superimposed thrombosis (75). Placing patients with CCD into perspective with the aforementioned hypercoagulation condition system, the association of both states of hypercoagulability can pose all patients with CCD at risk of cardiac thrombosis and PE. Therefore, it would be desirable that all coinfected and symptomatic patients require close medical attention irrespective of the severity of the diseases.

Anticoagulation is the cornerstone of the treatment of patients with CCHD at high-risk for thromboembolism: those with atrial fibrillation, cardiac thrombosis, previous thromboembolism, apical left ventricular aneurysm, or a risk score > 4 points (76). When coinfected with SARS-CoV-2, non-etheless, current data do not support the widespread use of anticoagulation in the outpatient setting to reduce the major adverse cardiovascular or pulmonary consequences associated with symptomatic but clinically stable conditions in patients without the abnormalities mentioned earlier, even in individuals with high-levels of D-dimer.

### Cardiac Arrhythmias and Sudden Cardiac Death

In a general population aged 60 years or older, the prevalence of premature ventricular contractions, supraventricular premature contractions, and supraventricular tachycardia is 8.4, 7.7, and 9.0%, respectively (77). However, in a non-elderly population, the prevalence of premature ventricular contractions and supraventricular premature contractions is 0.22 and 0.44%, respectively (78). The incidence of such arrhythmias is about 1% yearly (77). The prevalence of atrial fibrillation in a general population is 4.1% (77), whereas the incidence of atrial fibrillation is 0.75/100,000 inhabitants (2) or approximately 1% yearly (77).

The prevalence of sustained ventricular tachycardia (SVT) is low, usually affecting about 0.2% of the general population (79), whereas the incidence of SVT is about 13 cases/100,000 inhabitants (80). The prevalence of ventricular fibrillation can be surmised to be 6,3 cases/100,000 inhabitants (81). The incidence of ventricular fibrillation is about 1.83 per 10,000 inhabitants (82).

Palpitations can be observed in about 7% of hospitalized patients with COVID-19 (83). The overall prevalence of arrhythmias in hospitalized patients with COVID-19 seems to be 17%, which is much higher in critical patients than in non-critical patients (44 vs. 7%) (84). Several abnormalities underlie the appearance of arrhythmias in hospitalized patients with COVID-19, such as hypoxia, acid-base imbalance, electrolyte disturbance, and myocardial ischemia.

In a cohort of 140 patients with COVID-19 that underwent telemetry monitoring during hospitalization, seven (5%) developed ventricular tachycardia/ventricular fibrillation; all of these patients had metabolic, hypoxemic, or high vasopressor need (85). Elevated troponin serum levels may also be a harbinger of malignant arrhythmias in patients with this condition (83). Therefore, malignant arrhythmia *per se* does not seem to be the etiology of death in patients with critical or severe COVID-19.

On the other hand, palpitations can be the reason by which patients with COVID-19 search for medical assistance in the absence of any other symptoms (86). Therefore, arrhythmias can appear not only in hospitalized patients, but also early in the course of COVID-19. The prevalence, incidence, and clinical characteristics of arrhythmias in the setting of ambulatory patients or in those with long COVID-19 are not known at the present.

Premature ventricular contractions can be found in the resting electrocardiogram of about 7% of a population-based cohort of patients with CCD, whereas complex premature ventricular contractions (Lown classification grade III or higher) can be observed in 2% of them (43). In contrast, 43% of patients with CCD with CHF are found to have premature ventricular contractions (47). At 24h-Holter monitoring, non-sustained ventricular tachycardia affects approximately 21% of patients with CCHD and approximately 50% of patients with reduced LVEF (87), which appears to be 30 times more frequent than in those with non-Chagas disease heart failure (88).

The importance of detecting non-sustained ventricular tachycardia is that about 10% of patients with CCHD with this arrythmia subsequently present SVT (89), which shows a typical electrocardiographic pattern (90) and may degenerate into ventricular fibrillation in 24% of cases (91). Although the severity of premature ventricular contractions depends on the degree of ventricular function in patients with this condition, SVT or ventricular fibrillation can be observed in approximately 14% of patients with preserved LVEF who have recovered from cardiac arrest (91).

The association of complex premature ventricular contractions and moderately reduced LVEF overshadows the prognosis of patients with CCHD; the same, however, does not occur when the LVEF is severely reduced (< 30%) or normal (92). In a population-based cohort, the presence of complex premature ventricular contractions reduced survival to virtually zero percent at a 10-year follow up and was an independent predictor of mortality in patients with this condition (43).

SCD is a continuous threat for patients with CCD. In a general population of patients with CCD, the prevalence of SCD is about 29%, being the second leading cause of death following death from irreversible heart failure (93). In patients with CCHD, about 46% can experience SCD (43); in those with reduced LVEF, SCD is the mode of death more frequently found in patients with mild to moderate CHF than in those with advanced stages of the syndrome (94).

The annual incidence of SCD in patients with CCHD disease can be roughly estimated from 5 to 6% in a general population (27, 94) to 22% in patients from a referral center (43). A morphological study carried out in the first decade of this century showed that CCD is still the cause of SCD in approximately 5% of cases (95). SVT degenerating into ventricular fibrillation seems to be the main cause of SCD (91). Although advanced atrioventricular block was an important cause of SCD in patients with CCD before the era of pacemaker implantation, advanced atrioventricular block can rarely cause SCD nowadays (96).

Macro reentry at the His-Purkinje system can be the cause of SCD in patients with CCHD, inasmuch as there has been an association between left ventricular apical aneurysm and left anterior fascicular block in patients with this condition (97). However, the peculiarities of the underlying microscopic lesions—extensive myocardial inflammation intermingled with reparative fibrosis throughout the myocardium—form the environment to multiple sites of micro re-entry to cause SCD in patients with CCHD (96). The scar-associated reentry phenomenon observed in patients with SVT who underwent endocardial mapping for potential SVT treatment (98) lends support to this assumption.

In fact, when there is no myocardial fibrosis on cardiac magnetic resonance imaging, there is no aborted SCD or shock therapy with implantable cardioverter-defibrillator in patients with CCHD (99). In contrast, the presence of scar on magnetic resonance imaging predicted cardiovascular death or the appearance of SVT in a 34-median follow-up of patients with this condition (100). Furthermore, the amount of myocardial fibrosis on magnetic resonance imaging is higher in patients with malignant ventricular arrhythmia than in those without (101). In addition, the presence of two or more segments of delayed enhancement further stratifies the risk of SVT occurrence in patients with CCHD and myocardial fibrosis at magnetic resonance imaging (102). Therefore, myocardial fibrosis appears to play a central role in the pathogenesis of SCD in patients with CCHD.

Based on the clinical characteristics and the pathophysiological mechanism of arrhythmias mentioned earlier, it is plausible to consider that the association of COVID-19 and CCHD can predispose patients to developing malignant ventricular arrhythmias. Therefore, such patients must be telemonitored continuously when hospitalized, and metabolic abnormalities must be promptly corrected. Furthermore, high-risk outpatients with coinfection should be considered for screening of non-sustained ventricular tachycardia at 24-h Holter monitoring and 2-D echocardiography to assess LVEF, whenever possible. When they have long COVID-19, these patients should also be considered for magnetic resonance imaging examination to detect potential myocardial fibrosis, which must be treated according to guidelines.

## Conclusion

The association of COVID-19 and CCHD will probably overshadow the outcome of patients with CVD, i.e., those with CAD, CEVD, AHF/CHF, PE, and SCD. Despite the lack of guidelines on this subject, our suggestions may be of value to draw attention to doctors on the frontline of this deleterious association and to treat patients accordingly without any delay.

## Author Contributions

RBB, EB, and MN drafted the manuscript. RB, VI, and RF-D researched and discussed the manuscript. RBB and MN revised the final version. All authors have approved the manuscript in its final format.

## Funding

This work was supported by the University of Ribeirão Preto.

## Conflict of Interest

The authors declare that the research was conducted in the absence of any commercial or financial relationships that could be construed as a potential conflict of interest.

## Publisher's Note

All claims expressed in this article are solely those of the authors and do not necessarily represent those of their affiliated organizations, or those of the publisher, the editors and the reviewers. Any product that may be evaluated in this article, or claim that may be made by its manufacturer, is not guaranteed or endorsed by the publisher.

## Abbreviations

AHF: Acute heart failure
CAD: Coronary artery disease
CHF: Chronic heart failure
CCD: Chronic Chagas disease
CCHD: Chronic Chagas heart disease
CEVD: Cerebrovascular disease
CVD: Cardiovascular diseases
COVID-19: Coronavirus disease 2019
DALY: Disability-adjusted life years
LVEF: Left ventricular ejection fraction
PE: Pulmonary embolism
SARS-CoV-2: Severe acute respiratory syndrome coronavirus 2
SCD: Sudden cardiac death
STEMI: Acute myocardial infarction with elevated ST-segment
SVT: Sustained ventricular tachycardia.
